# The Effect of Caco-2 Cells on Sporulation and Enterotoxin Expression by Foodborne *Clostridium perfringens*

**DOI:** 10.3390/pathogens13060433

**Published:** 2024-05-21

**Authors:** Chao Wang, Tom Defoirdt, Andreja Rajkovic

**Affiliations:** 1 Laboratory of Food Microbiology and Food Preservation, Department of Food Technology, Safety and Health, Faculty of Bioscience Engineering, Ghent University, 9000 Ghent, Belgium; chao.wang@ugent.be; 2 Center for Microbial Ecology and Technology, Department of Biotechnology, Faculty of Bioscience Engineering, Ghent University, 9000 Ghent, Belgium; tom.defoirdt@ugent.be

**Keywords:** *Clostridium perfringens* enterotoxin, *cpe*, sporulation, host-pathogen interaction, Caco-2 cells

## Abstract

*Clostridium perfringens* enterotoxin (Cpe)-producing strains cause gastrointestinal infections in humans and account for the second-largest number of all foodborne outbreaks caused by bacterial toxins. The Cpe toxin is only produced during sporulation; this process might be affected when *C. perfringens* comes into contact with host cells. The current study determined how the *cpe* expression levels and spore formation changed over time during co-culture with Caco-2 cells (as a model of intestinal epithelial cells). In co-culture with Caco-2 cells, total *C. perfringens* cell counts first decreased and then remained more or less stable, whereas spore counts were stable over the whole incubation period. The *cpe* mRNA level in the co-culture with Caco-2 cells increased more rapidly than in the absence of Caco-2 cells (3.9-fold higher levels in coculture than in the absence of Caco-2 cells after 8 h of incubation). Finally, we found that *cpe* expression is inhibited by a cue released by Caco-2 cells (8.3-fold lower levels in the presence of supernatants of Caco-2 cells than in the absence of the supernatants after 10 h of incubation); as a consequence, the increased expression in co-culture with Caco-2 cells must be caused by a factor associated with the Caco-2 cells.

## 1. Introduction

*Clostridium perfringens* is a common pathogen causing histotoxic and intestinal infections in humans and animals [1,2]. Secreted toxins of *C. perfringens* play a vital role in host infection. The bacterium can produce 23 different toxins and many extracellular hydrolytic enzymes [3,4,5,6]. Strains belonging to this species are divided into seven types (from type A to G) based on the presence or absence of the main toxins [5,7]. *C. perfringens* enterotoxin (Cpe) is a toxin that causes food poisoning, and strains producing this toxin belong to *C. perfringens* type F.

Humans and animals are usually infected after coming into contact with foodborne *C. perfringens*, and the production of Cpe depends on sporulation [8]. The bacterial cells start an asymmetrical division to initiate the sporulation process and form a forespore and a mother cell. Forespores are released and mother cells lyse during the sporulation process, thereby releasing the toxin. After this, the toxin can exert its activity in the host intestine [9,10]. Thus far, research studies have primarily focused on sporulation media, growth characteristics, and the production of Cpe protein, but the *cpe* expression kinetics during sporulation have not been studied. 

The human epithelial cell line Caco-2 is widely used as a model for investigating the interaction between a toxin and the human host. Previous studies focused on the role of Cpe in activating the cell death pathway in the host or on investigating morphological effects of host exposure to Cpe [11,12,13,14,15]. Yasugi and coworkers found that type F *C. perfringens* strains only showed severe cytotoxicity during Cpe production and sporulation in co-culture with Caco-2 cells [16]. Also, they determined Cpe production in the presence of Caco-2 cells after 12 h by Western blot and reversed passive latex agglutination. However, *cpe* expression kinetics have not been reported during co-culture with host cells. Meanwhile, understanding *cpe* expression is vital to control *C. perfringens* enterotoxin production. Therefore, the aim of this study was to determine the impact of interaction with host cells on *cpe* expression and sporulation of *C. perfringens*, using Caco-2 cells as a model.

## 2. Materials and Methods

### 2.1. Bacterial Strain and Growth Conditions

The strain LMG 453, a *C. perfringens* type F food poisoning isolate [5,17], was obtained from the BCCM/LMG culture collection, stored at −75 °C on glass beads, and revived in 9 mL brain heart infusion broth (BHI, CM1135, Oxoid, Hampshire, UK) overnight at 37 °C in an anaerobic jar (HP0011A, Oxoid, Hampshire, UK) with an anaerobic indicator (BR0055, Oxoid, Hampshire, UK) and an anaerobic sachet (AN0035, Oxoid, Hampshire, UK). Overnight cultures were transferred into cold storage tubes (375418PK, Nunc, Thermo Scientific, Waltham, MA, USA) with 20% sterilized glycerol (CL00.0706, Chem-Lab, Zedelgem, Belgium) and stored at −75 °C until further use. To confirm the purity of *C. perfringens*, loops of the stock cultures were streaked on Tryptic Soy Agar (CM0131, Oxoid) plates and anaerobically incubated for 24 h at 37 °C, then stored at 4 °C as work stocks for up to 3 weeks. Trypticase peptone glucose yeast extract broth (50 g/L pancreatic digest of casein, 211921, BD; 5 g/L peptone, LP0034, Oxoid, Hampshire, UK; 20 g/L yeast extract, LP0021, Oxoid, Hampshire, UK; 4 g/L glucose, CL00.0710, Chem-Lab, Zedelgem, Belgium; 1 g/L sodium thioglycolate, STBJ0402, Sigma-Aldrich, St. Louis, MO, USA), and brain heart infusion agar (15 g/L agar LP0011, Oxoid, Hampshire, UK and 37 g/L BHI, CM1135, Oxoid, Hampshire, UK) were used to grow and count *C. perfringens*, respectively [18].

### 2.2. Sporulation of C. perfringens

A single colony of a stock culture was transferred to 10 mL of trypticase peptone glucose yeast extract broth, anaerobically incubated at 37 °C for 19 to 20 h, after which, 200 µL of the grown culture was transferred into a tube containing fresh trypticase peptone glucose yeast extract broth for the second overnight growth. The modified Duncan Strong medium (15 g/L proteose peptone, LP0085, Oxoid, Hampshire, UK; 4 g/L yeast extract, LP0021, Oxoid, Hampshire, UK; 1 g/L sodium thioglycolate, STBJ0402, Sigma-Aldrich, St. Louis, MO, USA; 10 g/L sodium phosphate dibasic, BCBV5946, Sigma-Aldrich; 4 g/L raffinose, BCCC1674, Sigma-Aldrich) was used for *C. perfringens* sporulation. The pH was adjusted to 7.8 ± 0.1 at room temperature before use, and the medium was prepared within three days before use [18,19]. Ten per cent by volume of a grown *C. perfringens* culture was transferred into a sterilized modified Duncan Strong medium and anaerobically incubated at 37 °C to initiate sporulation [19]. All samples were heated to 70.5 °C in a water bath (GD 120, Grant Instruments Ltd., Cambridge, UK) for 20 min to inactivate vegetative *C. perfringens* cells and then promptly cooled on ice water for at least 3 min. Then, samples were serially diluted in peptone physiological solution (1 g/L peptone; 8.5 g/L NaCl, S9888, Sigma-Aldrich) and spread-plated on brain heart infusion agar in duplicate, after which the plates were anaerobically incubated for 24 h at 37 °C. The experiment was repeated three times.

### 2.3. Caco-2 Cell Culture

Caco-2 cells (ATCC HTB37) were obtained from the American Type Culture Collection. The cells were cultivated in Dulbecco’s Modified Eagle Medium supplemented with GlutaMAX (10569010, Life Technologies Limited, Renfrew, UK), 10% fetal bovine serum (F7524, Sigma-Aldrich), 1% non-essential amino acids (11140035, Life Technologies Limited, Renfrew, UK), and 1% penicillin/streptomycin (15140122, Life Technologies Limited, Renfrew, UK). Caco-2 cells were grown aerobically at 37 °C in a humidified environment containing 10% CO_2_ and subcultured by trypsinization at 80% confluency. The medium was changed every two or three days, and the cells were passaged less than 50 times.

### 2.4. Collection of Caco-2 Cell Supernatants

Caco-2 cells were grown in Dulbecco’s Modified Eagle Medium until they reached 80% confluency. Then, the cells were washed once with phosphate-buffered saline (PBS) and then incubated in PBS without Ca^2+^, Mg^2+^ (14190144, Gibco, Life Technologies Europe B.V., Bleiswijk, The Netherlands) for 24 h aerobically at 37 °C in a humidified environment containing 10% CO_2_. The cell suspension was collected into a 50 mL falcon tube (02-527-3001, nerbe plus, Winsen (Luhe), Germany) and centrifuged at 1000× *g* for 3 min at room temperature. Then, the supernatants were filtered through a 0.22 µM filter (A35150, Novolab NV, Geraardsbergen, Belgium), and 10 mL aliquots were stored at −20 °C until further use.

### 2.5. Interaction between C. perfringens and Caco-2 Cells or Caco-2 Cell Supernatants

Caco-2 cells were seeded at a density of 2 × 10^5^ cells/well in PBS in 6-well plates (657160, Greiner Bio-One, Vilvoorde, Belgium) and incubated aerobically for 24 h at 37 °C in a humidified environment containing 10% CO_2_ to allow the cells to adhere. *C. perfringens* cells were rinsed twice with PBS before further use. Subsequently, 2 mL volumes of *C. perfringens* in phosphate-buffered saline were added per well, with a Caco-2 cell-to-bacterium ratio of 1:100. As controls, *C. perfringens* cells in PBS or Caco-2 cell supernatants were added to empty wells. The suspensions were incubated for 6-12 h at 37 °C in a humidified environment containing 10% CO_2_. After incubation, *C. perfringens* were collected and separated into two groups using cell scrapers (541070, Greiner Bio-One, Vilvoorde, Belgium). One group was heated to inactivate vegetative *C. perfringens* cells, while the other was kept at room temperature as a control. *C. perfringens* cell densities were determined by spread-plating on brain heart infusion agar and anaerobic incubation for 24 h at 37 °C. Experiments were done with three biological repeats.

### 2.6. Determination of C. perfringens Enterotoxin (cpe) mRNA Levels

*cpe* mRNA levels were determined as described recently [20]. Briefly, total RNA was extracted from *C. perfringens* with/without Caco-2 cells using the RNeasy Protect Bacteria Mini Kit (74524, Qiagen, Hilden, Germany). Genomic DNA was removed from total RNA using the RapidOut DNA Removal Kit (K2981, Thermo Scientific, Dreieich, Germany). The concentration of extracted total RNA was measured using a Quantus™ Fluorometer (E6150, Promega, Madison, WI, USA). Following that, 1 µg of total RNA was utilized to synthesize cDNA using the RevertAid H Minus First Strand cDNA Synthesis Kit (K1632, Thermo Scientific, Dreieich, Germany). All processes were carried out according to the manufacturer’s instructions.

The mRNA levels of *cpe* were quantified by reverse transcription quantitative PCR (RT-qPCR). Glyceraldehyde 3-phosphate dehydrogenase (*gapdh*) was used as the reference gene. The RT-qPCR mixture contained 12.5 μL PowerUp™ SYBR™ Green Master Mix (A25742, Applied Biosystems, Waltham, MA, USA), 2 μL cDNA, 500 nM forward and reverse primers (Table 1), and nuclease-free water (129115, Qiagen, Hilden, Germany) to a total volume of 25 μL.

**Table 1:** Primers used in this study.

Primer	Sequence (5′-3′)	Amplicon Size (bp)	Reference
cpe F	GGAGATGGTTGGATATTAGG	233	[21]
cpe R	GGACCAGCAGTTGTAGATA		
GAPDH F	AACAAGAGAACCTTTAGGGG	123	[20]
GAPDH R	GTAGCAGGTTTAAGCACAAC		

The RT-qPCR was completed with an Applied Biosystems 7300 real-time PCR system (Applied Biosystem, Waltham, MA, USA). The conditions of RT-qPCR were as follows: 95 °C for 10 min followed by 40 cycles of 95 °C for 15 s, 55 °C for 60 s, 72 °C for 60 s, and 1 cycle of 95 °C for 15 s, 60 °C for 60 s, 95 °C for 15 s and 60 °C for 15 s as a final extension. All experiments included at least two biological replicates for RNA extraction and three technical replicates for RT-qPCR for each sample. The relative *cpe* expression level was calculated using the comparative threshold cycle technique (2^−ΔΔCt^) [22,23].

### 2.7. Statistical Analyses

Data analysis was performed and generated using SPSS Statistics 26.0 software (IBM SPSS Statistics, NY, USA) and graphs were constructed using GraphPad Prism 9.0 (GraphPad Software, San Diego, CA, USA). All data were expressed as mean ± standard deviations. The lognormality of all enumeration and RT-qPCR data were checked by the Anderson–Darling test, D’Agostino–Pearson omnibus test, Shapiro–Wilk test, and QQ plot. Differences in *cpe* mRNA levels over time in the modified Duncan Strong medium were assessed using one-way ANOVA, followed by Dunnett’s post hoc test. Two-way ANOVA, followed by Tukey’s post hoc test, was used to analyze the differences between the *cpe* expression of *C. perfringens* incubated in the presence of Caco-2 cells or Caco-2 cell supernatants. Differences were considered significant if the *p* value was less than 0.05.

## 3. Results

### 3.1. Growth, Sporulation, and cpe Expression of C. perfringens in Modified Duncan Strong Medium

**Figure 1:**
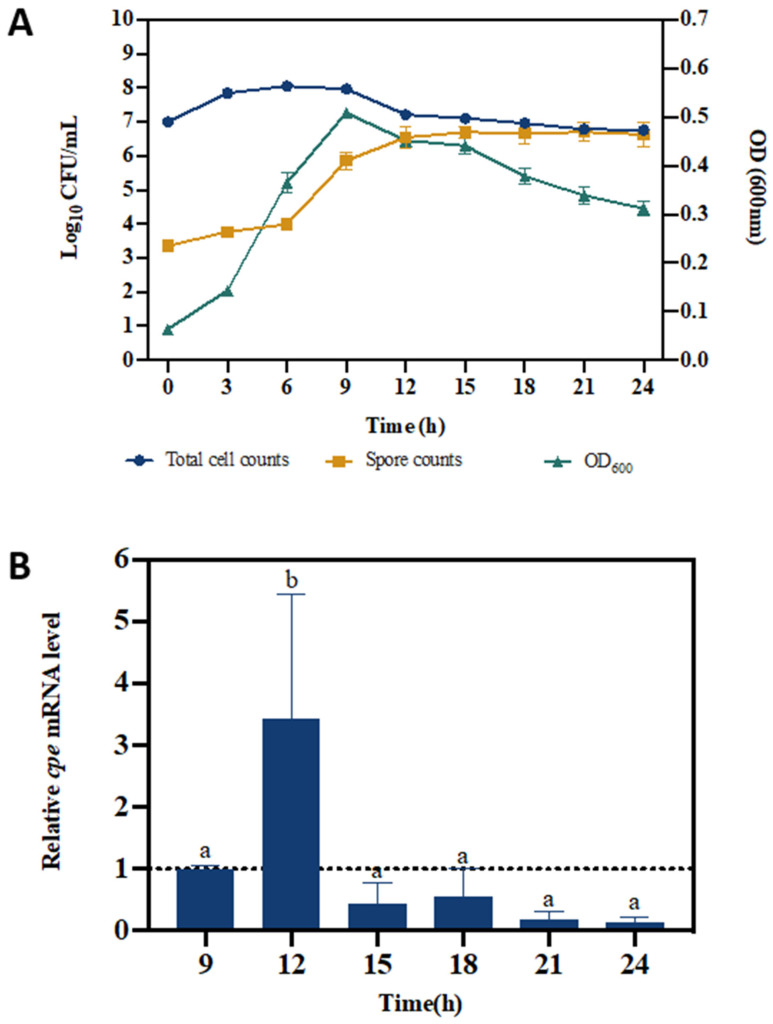
Growth, sporulation, and *cpe* expression of *C. perfringens* in modified Duncan Strong medium. (**A**) Total *C. perfringens* cell counts, spore counts, and OD600 measurements. (**B**) Relative *cpe* mRNA levels of *C. perfringens* during incubation in modified Duncan Strong medium. The expression level at 9 h was set at 1 and the levels at the other time points were normalized accordingly using the 2^−ΔΔCT^ method. The *gapdh* gene was used as the reference gene. The error bars represent the standard deviations of 3 biological replicates. Different letters indicate significant differences in *cpe* mRNA levels (one-way ANOVA, followed by Dunnett’s post hoc test; *p* < 0.05).

*C. perfringens* cell counts in modified Duncan Strong medium were determined both before and after heat treatment in order to distinguish between total cell counts and spore counts, respectively. Total cell counts increased with one log unit in the first three hours, then remained constant for 6 h, and then decreased again with one log unit (Figure 1A). OD600 measurements reflected this trend, with a maximum obtained after 9 h incubation and a gradual decrease thereafter. Spore counts, on the other hand, increased with more than 2 log units between 6 and 12 h of incubation, after which the level remained constant. From 12 h onwards, virtually all *C. perfringens* were spores.

The relative *cpe* mRNA level of *C. perfringens* peaked after 12 h of incubation (3.4 times higher than the level at 9 h), after which, it gradually decreased again (Figure 1B). These results demonstrate that our methodology enables us to determine sporulation and *cpe* expression in *C. perfringens*.

### 3.2. Survival, Sporulation, and cpe Expression of the C. perfringens Enterotoxin (cpe) Gene during Contact with Caco-2 Cells

**Figure 2:**
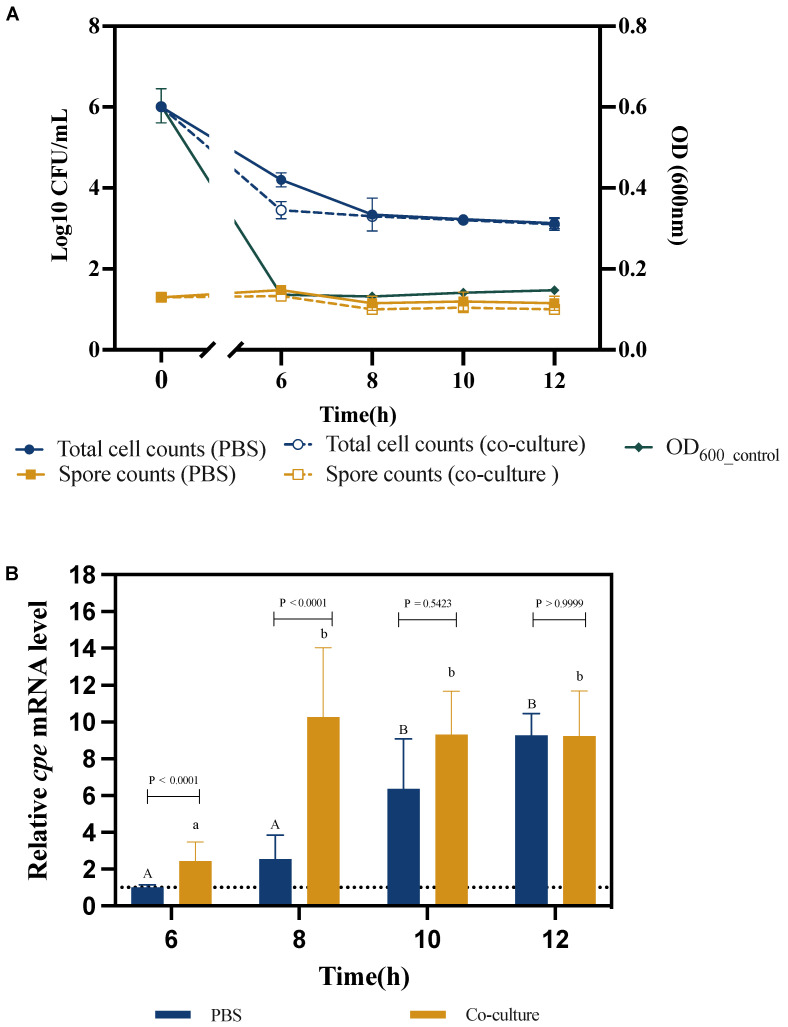
Survival, sporulation, and *cpe* expression of *C. perfringens* in co-culture with Caco-2 cells compared to the PBS control treatment. (**A**) Total *C. perfringens* cell counts, spore counts and OD600 over time. (**B**) Relative *cpe* mRNA levels of *C. perfringens* with/without Caco-2 cells. The expression level in the PBS treatment after 6 h was set at 1, and the expression levels for all treatments at the other time points were normalized accordingly using the 2^−ΔΔCT^ method. The *gapdh* gene was used as the reference gene. The initial ratio *C. perfringens*: Caco-2 cells in the co-culture was 100:1 (MOI = 100). Experiments were conducted in three biological replicates and the error bars present the standard deviations. Different letters (capitals for the PBS treatment; small letters for the co-culture) indicate significant differences in *cpe* mRNA levels at different time points within each treatment (Two-way ANOVA, followed by Tukey’s post hoc test; *p* < 0.05). *p* values for the comparison between PBS and co-culture at each time point are also indicated.

In this experiment, we aimed to investigate the impact of exposure of *C. perfringens* to Caco-2 cells on sporulation and *cpe* mRNA levels. The total cell counts of *C. perfringens* and the OD600 values gradually decreased over time, with a reduction of over 2 log units in total *C. perfringens* cell counts during the first 8 h and a decrease of more than 0.4 OD600 units during the first 6 h (Figure 2A). The *C. perfringens* total cell counts decreased a bit more rapidly in co-culture than in the PBS control but reached the same level after 8 h. Spore counts, on the other hand, remained more or less stable over the incubation and did not differ between co-culture and PBS control.

We further determined the impact of co-culture with Caco-2 cells on *cpe* mRNA levels. In the PBS control treatment, the *cpe* mRNA level increased over time and was significantly different from the level at the 6 h time point after 10 h of incubation (6-fold higher than the level at the 6 h time point; Figure 2B). The *cpe* mRNA level in the coculture increased more rapidly and reached its maximum after 8 h already (9.8-fold higher than in the PBS control after 6 h), and at that time point, the *cpe* mRNA level in the coculture was significantly different from that in the PBS control (3.9-fold higher levels).

### 3.3. Survival, Sporulation and cpe Expression of C. perfringens in Caco-2 Cell Supernatants

**Figure 3:**
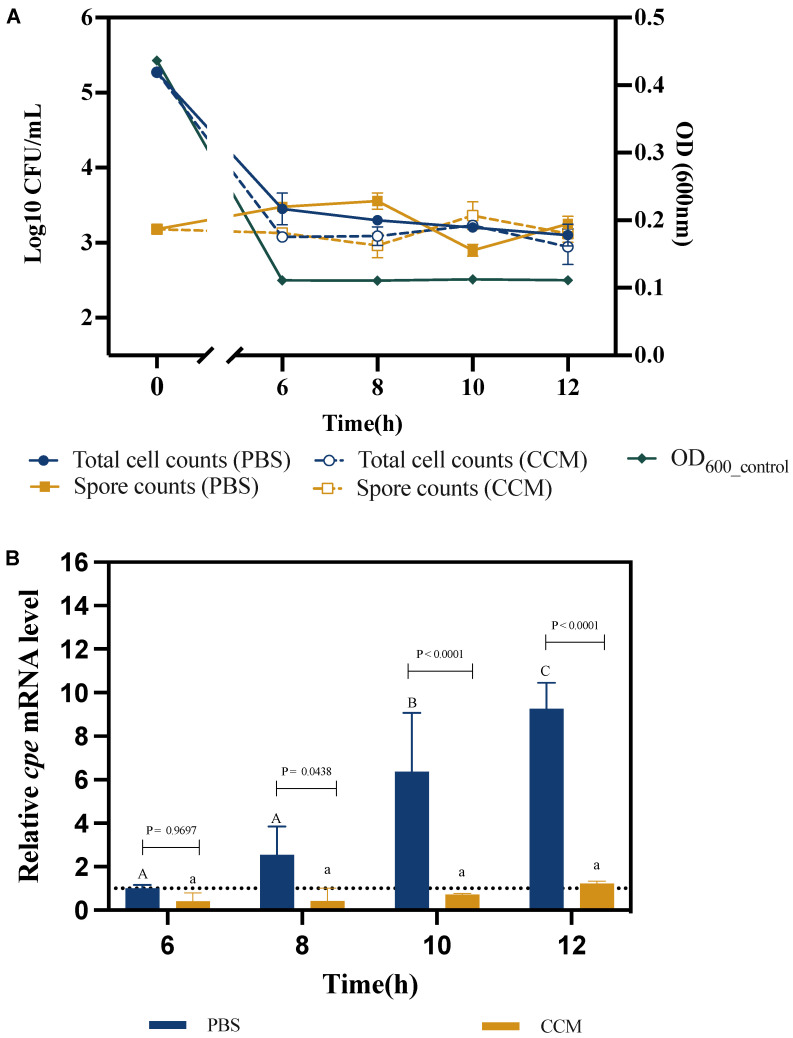
Survival, sporulation, and *cpe* expression of *C. perfringens* in Caco-2 cell supernatants (CCM) compared to the PBS control treatment. (**A**) Total *C. perfringens* cell counts, spore counts and OD600 over time. (**B**) Relative *cpe* mRNA levels. The expression level in the PBS treatment after 6 h was set at 1, and the expression levels for all treatments at the other time points were normalized accordingly using the 2^−ΔΔCT^ method. The *gapdh* gene was used as the reference gene. Experiments were done in three biological replicates and the error bars present the standard deviations. Different letters (capitals for the PBS treatment; small letters for the co-culture) indicate significant differences in *cpe* mRNA levels at different time points within each treatment (Two-way ANOVA, followed by Tukey’s post hoc test; *p* < 0.05). *p* values for the comparison between PBS and co-culture at each time point are also indicated.

In the last experiment, we aimed to determine whether the difference in *cpe* expression observed in co-culture with Caco-2 cells was mediated by a cue released by the Caco-2 cells. To this end, we determined the survival, sporulation, and *cpe* expression of *C. perfringens* in the presence of Caco-2 cell supernatants. Similar to what we observed in the previous experiment, the total *C. perfringens* cell counts and OD600 decreased at the beginning of the experiment and remained constant thereafter (Figure 3A). Also similar to the previous experiment, there was no clear trend in spore counts.

Similar to the previous experiment, the relative *cpe* mRNA levels increased over time in the PBS control treatment (Figure 3B). However, the *cpe* mRNA levels of *C. perfringens* incubated in Caco-2 cell supernatants were significantly lower than those in the PBS control at all sampling points, with a maximal difference at the 10 h sampling point (8.3-fold difference).

**Figure 4:**
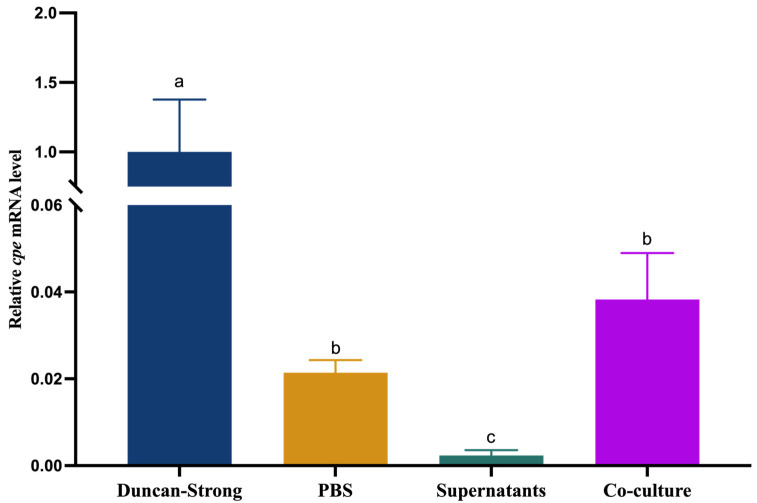
Relative *cpe* mRNA level of *C. perfringens* in different media (modified Duncan Strong medium, PBS, Caco-2 cell supernatants, and co-culture with Caco-2 cells) after 12 h of incubation. The initial ratio *C. perfringens*: Caco-2 cells in the co-culture was 100:1 (MOI = 100). The expression level in the modified Duncan Strong medium was set at 1 and the levels in the other treatments were normalized accordingly using the 2^−ΔΔCT^ method. The *gapdh* gene was used as the reference gene. Experiments were conducted with at least two biological replicates and the error bars represent standard deviations. Different letters indicate significant differences in *cpe* mRNA levels between treatments (Two-way ANOVA, followed by Tukey’s post hoc test; *p* < 0.05).

We finally compared the *cpe* mRNA level in PBS, in co-culture with Caco-2 cells, and in the presence of supernatants of Caco-2 cells to that in modified Duncan Strong medium and found that it was still about 25-fold lower in co-culture with Caco-2 cells than in modified Duncan Strong medium (Figure 4).

## 4. Discussion

In this study, we aimed to determine the impact of interaction with host cells on sporulation and *cpe* expression in *C. perfringens*. We first validated our methodology using a modified Duncan Strong medium. The modified Duncan Strong medium is the most common medium for the cultivation and induction of sporulation of *C. perfringens*. Although the sporulation ability and *cpe* expression of *C. perfringens* may vary from strain to strain, the strain used in this study (LMG 453) was sporulating well in this medium (Figure 1A). The spore formation did not change within the first 6 h of incubation, after which it increased dramatically (more than 100-fold difference). Similarly, De Jong and coworkers reported a significant increase in the number of spores of *C. perfringens* after 5 h in a modified Duncan Strong medium [19]. Sporulation is a sign for *C. perfringens* to produce the enterotoxin, and *cpe* mRNA was indeed the highest at the moment when most *C. perfringens* cells had sporulated (3.4-fold increase), and they decreased again thereafter. Similarly, Melville and coworkers found that the *cpe* mRNA of strain NCTC 10240 reached the highest level after 10 h of incubation in Duncan Strong medium, and at the same time point, the enterotoxin protein concentration also achieved the maximal level [24]. Our results indicated that the methodology we used enabled us to detect sporulation and *cpe* expression in the *C. perfringens* strain we used and, thus, could be used in further experiments.

We subsequently aimed to investigate the impact of coming into contact with human cells on sporulation and *cpe* expression. We used Caco-2 cells as a model as this cell line has been widely used as a model of intestinal epithelial cells [25]. We found that contact with Caco-2 cells did not induce sporulation in our strain. Rather, spore counts remained stable during incubation in the presence of Caco-2 cells (Figure 2A), whereas total *C. perfringens* cell counts sharply decreased (more than 100-fold) during the first hours of incubation, and this was also observed in the control, in which *C. perfringens* was incubated in PBS under the same conditions (aerobic incubation at 37 °C in a humidified environment containing 10% CO_2_). This finding reflects those of Paredes-Sabja and Sarker, who found that the total cell number of *C. perfringens* strain F4969 decreased during aerobic incubation in the presence of 5% CO_2_ in cell growth medium [26]. They also showed that the number of heat-resistant spores remained constant from 0.5 h to 24 h for three strains cocultured with Raw 264.7 cells under the same conditions. As *C. perfringens* is an anaerobic bacterium, the presence of oxygen was likely responsible for the decrease in total cell counts. Indeed, previous studies have demonstrated that although oxygen itself does not cause *C. perfringens* death immediately, it results in the formation of hydroxyl radicals, hydrogen peroxide, or superoxide radicals, which are toxic to vegetative cells of *C. perfringens* [27,28]. This can explain why the total number of *C. perfringens* cells decreased during the first hours of exposure to Caco-2 cell growth conditions.

We also measured the *cpe* mRNA levels during incubation under Caco-2 cell culture conditions and found that they increased over time. Remarkably, the *cpe* mRNA levels increased faster when cocultured with Caco-2 cells than in the PBS control (5-fold difference in *cpe* mRNA levels after 8 h of incubation—Figure 2B). This result is in agreement with the findings of Vidal and coworkers, who showed that ß toxin and perfringolysin O production were upregulated in type C *C. perfringens* when the bacterium was incubated in the presence of Caco-2 cells [29]. Interestingly, Yasugi and coworkers demonstrated the presence of Cpe in the supernatants of cocultures of type A *C. perfringens* and Caco-2 cells at 24 h by Western blot [16]. Although this result indicated that Cpe was produced at 24 h, the kinetics of Cpe production were not studied; therefore, our study fills this knowledge gap by showing that *cpe* expression is induced relatively early (i.e., within 8 h) during contact with Caco-2 cells.

It has been shown before that mammalian cells can release cues, such as hormones, which can affect toxin production in bacterial pathogens [30,31]. As Caco-2 cells are also able to produce hormones [32], we hypothesized that the increased expression of *cpe* might be caused by such a cue released by the Caco-2 cells. In order to investigate this possibility, cell-free supernatants of Caco-2 cells were collected and *C. perfringens* was subsequently exposed to it. In contrast to our expectations, *cpe* expression was inhibited rather than induced in these supernatants (Figure 3B). This indicates that the *cpe* expression in co-culture with Caco-2 cells was inhibited rather than induced by cues released from Caco-2 cells, and therefore, the induction of *cpe* expression in co-culture was caused by a factor that is associated with the Caco-2 cells rather than a secreted cue. We very recently reported that mucins strongly increase the expression of *cpe* in *C. perfringens* [20]. Caco-2 cells also produce mucins [33] and consequently, we hypothesize that mucins secreted by and attached to the Caco-2 cells might have been responsible for the increased expression of *cpe* during coculture of *C. perfringens* with Caco-2 cells.

Together, our results indicate that *cpe* expression is inhibited by a cue released by Caco-2 cells and induced by a factor associated with the cells, and in co-culture with Caco-2 cells, the inhibition is overruled by induction. This suggests that *cpe* expression is activated in the gut when *C. perfringens* is close to the epithelium, whereas it is inhibited when the bacterium is located farther away from the epithelium. The cell-associated factor inducing *cpe* expression might be mucin, whereas the identification of the released cue that inhibits *cpe* expression needs further research.

## Data Availability

The data associated with this article will be shared upon reasonable request to the corresponding author.

## References

[1] Collie R.E., Kokai-Kun J.F., McClane B.A. (1998). Phenotypic characterization of enterotoxigenic *Clostridium perfringens* isolates from non-foodborne human gastrointestinal diseases. Anaerobe.

[2] McClane B.A., Chakrabarti G. (2004). New insights into the cytotoxic mechanisms of *Clostridium perfringens* enterotoxin. Anaerobe.

[3] Chen J.M., McClane B.A., Chen J.M., McClane B.A. (2015). Characterization of *Clostridium perfringens* TpeL toxin gene carriage, production, cytotoxic contributions, and trypsin sensitivity. Infect. Immun..

[4] Dabrowski S., Staat C., Zwanziger D., Sauer R.-S.S., Bellmann C., Gunther R., Krause E., Haseloff R.F., Rittner H., Blasig I.E. (2015). Redox-sensitive structure and function of the first extracellular loop of the cell-cell contact protein claudin-1: Lessons from molecular structure to animals. Antioxid. Redox Signal..

[5] Kiu R., Hall L.J. (2018). An update on the human and animal enteric pathogen *Clostridium perfringens*. Emerg. Microb. Infect..

[6] Shrestha A., Uzal F.A., McClane B.A. (2018). Enterotoxic Clostridia: *Clostridium perfringens* enteric diseases. Microbiol. Spectr..

[7] Rajkovic A., Jovanovic J., Monteiro S., Decleer M., Andjelkovic M., Foubert A., Beloglazova N., Tsilla V., Sas B., Madder A. (2020). Detection of toxins involved in foodborne diseases caused by Gram-positive bacteria. Compr. Rev. Food Sci. Food Saf..

[8] Paredes-Sabja D., Sarker M.R. (2011). Host serum factor triggers germination of *Clostridium perfringens* spores lacking the cortex hydrolysis machinery. J. Med. Microbiol..

[9] Harry K.H., Zhou R., Kroos L., Melville S.B. (2009). Sporulation and enterotoxin (CPE) synthesis are controlled by the sporulation-specific sigma factors SigE and SigK in *Clostridium perfringens*. J. Bacteriol..

[10] Zhao Y.L., Melville S.B. (1998). Identification and characterization of sporulation-dependent promoters upstream of the enterotoxin gene (*cpe*) of *Clostridium perfringens*. J. Bacteriol..

[11] Chakrabarti G., Zhou X., McClane B.A. (2003). Death pathways activated in CaCo-2 cells by *Clostridium perfringens* enterotoxin. Infect. Immun..

[12] Garcia J.P., Li J., Shrestha A., Freedman J.C., Beingesser J., McClane B.A., Uzal F.A. (2014). *Clostridium perfringens* type A enterotoxin damages the rabbit colon. Infect. Immun..

[13] Low L.-Y., Harrison P.F., Gould J., Powell D.R., Choo J.M., Forster S.C., Chapman R., Gearing L.J., Cheung J.K., Hertzog P. (2018). Concurrent host-pathogen transcriptional responses in a *Clostridium perfringens* murine myonecrosis infection. mBio.

[14] Miyakawa M.E.F., Creydt V.P., Uzal F.A., McClane B.A., Ibarra C. (2005). *Clostridium perfringens* enterotoxin damages the human intestine in vitro. Infect. Immun..

[15] Singh U., Van Itallie C.M., Mitic L.L., Anderson J.M., McClane B.A. (2000). CaCo-2 cells treated with *Clostridium perfringens* enterotoxin form multiple large complex species, one of which contains the tight junction protein occludin. J. Biol. Chem..

[16] Yasugi M., Sugahara Y., Hoshi H., Kondo K., Talukdar P.K., Sarker M.R., Yamamoto S., Kamata Y., Miyake M. (2015). In vitro cytotoxicity induced by *Clostridium perfringens* isolate carrying a chromosomal *cpe* gene is exclusively dependent on sporulation and enterotoxin production. Microb. Pathog..

[17] Löffler A., Labbe R.G. (1983). Intracellular proteases during sporulation and enterotoxin formation by *Clostridium perfringens* type A. Curr. Microbiol..

[18] Atlas R.M. (2010). Handbook of Microbiological Media.

[19] De Jong A.E.I., Beumer R.R., Rombouts F.M. (2002). Optimizing sporulation of *Clostridium perfringens*. J. Food Prot..

[20] Wang C., Defoirdt T., Rajkovic A. (2024). The impact of indole and mucin on sporulation, biofilm formation and enterotoxin production in foodborne *Clostridium perfringens*. J. Appl. Microbiol..

[21] Meer R.R., Songer J.G. (1997). Multiplex polymerase chain reaction assay for genotyping *Clostridium perfringens*. Am. J. Vet. Res..

[22] Livak K.J., Schmittgen T.D. (2001). Analysis of relative gene expression data using real-time quantitative PCR and the 2^−ΔΔCT^ method. Methods.

[23] Yang Q., Defoirdt T. (2015). Quorum sensing positively regulates flagellar motility in pathogenic *Vibrio harveyi*. Environ. Microbiol..

[24] Melville S.B., Labbe R., Sonensheinl A.L. (1994). Expression from the *Clostridium perfringens cpe* promoter in *C. perfringens* and *Bacillus subtilis*. Infect. Immun..

[25] Sambuy Y., De Angelis G., Ranaldi M.L., Scarino A., Stammati F., Zucco F. (2005). The Caco-2 cell line as a model of the intestinal barrier: Influence of cell and culture-related factors on Caco-2 cell functional characteristics. Cell Biol. Toxicol..

[26] Paredes-Sabja D., Sarker M.R. (2012). Interactions between *Clostridium perfringens* spores and Raw 264.7 macrophages. Anaerobe.

[27] Briolat V., Reysset G. (2002). Identification of the *Clostridium perfringens* genes involved in the adaptive response to oxidative stress. J. Bacteriol..

[28] Jean D., Briolat V., Reysset G. (2004). Oxidative stress response in *Clostridium perfringens*. Microbiology.

[29] Vidal J.E., Ohtani K., Shimizu T., McClane B.A. (2009). Contact with enterocyte-like Caco-2 cells induces rapid upregulation of toxin production by *Clostridium perfringens* type C isolates. Cell. Microbiol..

[30] Kendall M.M., Sperandio V. (2016). What a dinner party! Mechanisms and functions of interkingdom signaling in host-pathogen associations. mBio.

[31] Li Z., Quan G., Jiang X., Yang Y., Ding X., Zhang D., Wang X., Hardwidge P.R., Ren W., Zhu G. (2018). Effects of metabolites derived from gut microbiota and hosts on pathogens. Front. Cell. Infect. Microbiol..

[32] Song W.Y., Aihara Y., Hashimoto T., Kanazawa K., Mizuno M. (2015). (−)-Epigallocatechin-3-gallate induces secretion of anorexigenic gut hormones. J. Clin. Biochem. Nutr..

[33] Van Klinken B.J., Oussoren E., Weenink J.J., Strous G.J., Büller H.A., Dekker J., Einerhand A.W. (1996). The human intestinal cell lines Caco-2 and LS174T as models to study cell-type specific mucin expression. Glycoconj. J..

